# 6-Allyl-8-meth­oxy-3-phenyl-3,4-dihydro-2*H*-benzo[*e*][1,3]oxazine

**DOI:** 10.1107/S1600536811027474

**Published:** 2011-07-16

**Authors:** Jing Zhu, Zhi-Dong Ren, Yang Liu, Lei Zhao, Yong Wu

**Affiliations:** a School of Chemistry and Chemical Engineering, Henan University of Technology, Zhengzhou 450001, People’s Republic of China

## Abstract

In the title compound, C_18_H_19_NO_2_, the allyl group is disordered over two sets of sites [occupancy ratio 0.662 (4):0.338 (4)]. The dihedral angle between the phenyl and benzene rings is 87.44 (10)°. The oxazinane ring adopts a sofa conformation.

## Related literature

For similar heterocyclic compounds, see: Chen *et al.* (2007 ▶); Kiskan *et al.* (2007 ▶); Liu *et al.* (2007 ▶); Ran & Gu (2011 ▶); Sawaryn *et al.* (2010 ▶); Su *et al.* (2005 ▶). For puckering parameters, see: Cremer & Pople (1975 ▶).
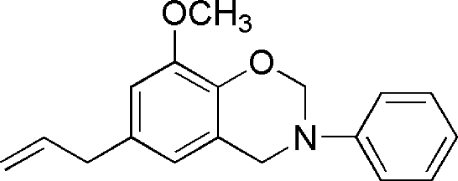


## Experimental

### 

#### Crystal data

C_18_H_19_NO_2_
*M* *_r_* = 281.34
Triclinic, 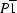

*a* = 8.4087 (5) Å
*b* = 9.4852 (5) Å
*c* = 10.7735 (7) Å
α = 99.193 (5)°
β = 98.900 (5)°
γ = 115.476 (6)°
*V* = 741.30 (9) Å^3^
*Z* = 2
Cu *K*α radiation
μ = 0.65 mm^−1^
*T* = 291 K
0.20 × 0.18 × 0.18 mm

#### Data collection

Oxford Diffraction Xcalibur, Eos, Gemini diffractometer
Absorption correction: multi-scan (*CrysAlis PRO*; Oxford Diffraction, 2010 ▶) *T*_min_ = 0.659, *T*_max_ = 1.000
5471 measured reflections
2644 independent reflections
2282 reflections with *I* > 2σ(*I*)
*R*_int_ = 0.015

#### Refinement

*R*[*F* ^2^ > 2σ(*F* ^2^)] = 0.047
*wR*(*F* ^2^) = 0.137
*S* = 1.05
2644 reflections
190 parameters
H-atom parameters constrained
Δρ_max_ = 0.48 e Å^−3^
Δρ_min_ = −0.50 e Å^−3^

### 

Data collection: *CrysAlis PRO* (Oxford Diffraction, 2010); cell refinement: *CrysAlis PRO*; data reduction: *CrysAlis PRO*; program(s) used to solve structure: *SHELXS97* (Sheldrick, 2008 ▶); program(s) used to refine structure: *SHELXL97* (Sheldrick, 2008 ▶); molecular graphics: *OLEX2* (Dolomanov *et al.*, 2009 ▶); software used to prepare material for publication: *OLEX2*.

## Supplementary Material

Crystal structure: contains datablock(s) I, global. DOI: 10.1107/S1600536811027474/bx2360sup1.cif

Supplementary material file. DOI: 10.1107/S1600536811027474/bx2360Isup3.cml

Structure factors: contains datablock(s) I. DOI: 10.1107/S1600536811027474/bx2360Isup2.hkl

Additional supplementary materials:  crystallographic information; 3D view; checkCIF report

## Figures and Tables

**Fig. 1.**
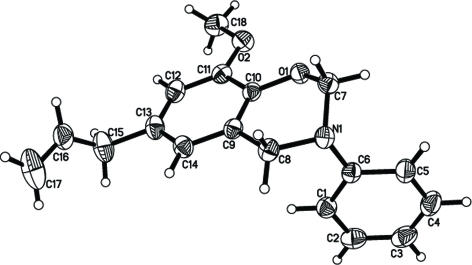
Molecular structure of (I), with the atom-labeling scheme. Displacement ellipsoids are drawn at the 30% probability level. Only the major occupied component is shown.

## supplementary crystallographic information

### Comment

Benzo[*e*][1,3]oxazines, which can be cured *via* a thermal ring opening reaction to construct an analogous phenolic structure characterized by a Mannich base bridge (–CH2—NR—CH2), are an important class of heterocycles (Su *et al.*, 2005, Kiskan *et al.*, 2007; Liu *et al.*, 2007, Ran & Gu, 2011, Sawaryn *et al.*, 2010). The title compound (I) was prepared by reaction of aniline, formaldehyde and 4-allyl-2-methoxyphenol. We report here the crystal structure of (I) .

The molecular structure of title compound (I) is showing in Fig. 1. The dihedral angle between the phenyl and benzene rings is 87.44 (10)° and this value is longer than similar compound reported by Chen *et al.*, 2007. The allyl group was refined using a disorder model with an occupancy ratio of 0.662 (4):0.338 (4). The oxazinane ring of the benzoxazine moiety adopts the sofa conformation, with the puckering parameters q_2 _= 0.3505 (16) Å and φ = 272.3 (3)° (Cremer & Pople, 1975) .

### Experimental

Aniline (0.05 mol), formaldehyde (0.1 mol), 4-allyl-2-methoxyphenol (0.05 mol) and 1,4-dioxine (50 ml) were introduced into a 250 ml flask, and the mixtures were stirred at 60 °C for 5 h, then condensed by rotary evaporators (35 °C), a red mucus was got and set at 15 °C for a few hour. The title compound was precipitated out in the meantime and washed by methanol. Colourless crystals suitable for X-ray diffraction analysis were obtained by recrystallization from methanol. And then the crystal of title compound was mounted in inert oil and transferred to the cold gas stream of the diffractometer.

### Refinement

All H atoms were placed in geometrically calculated positions with C—H = 0.93 Å and were refined isotropic with *U*_iso_(H) = 1.2*U*_eq_(C) of parent atom using a riding model.

Atoms C16 of the allyl group is disordered and was refined using a disorder model with site occupancy factors of 0.662 (4) and 0.338 (4). The corresponding bond distances in the disordered groups were restrained to be equal.

### Crystal data

**Table:**

C_18_H_19_NO_2_	*Z* = 2
*M**_r_* = 281.34	*F*(000) = 300
Triclinic, *P*1	*D*_x_ = 1.260 Mg m^−^^3^
*a* = 8.4087 (5) Å	Cu *K*α radiation, λ = 1.5418 Å
*b* = 9.4852 (5) Å	Cell parameters from 3645 reflections
*c* = 10.7735 (7) Å	θ = 4.3–71.9°
α = 99.193 (5)°	µ = 0.65 mm^−^^1^
β = 98.900 (5)°	*T* = 291 K
γ = 115.476 (6)°	Prismatics, colourless
*V* = 741.30 (9) Å^3^	0.20 × 0.18 × 0.18 mm

### Data collection

**Table:**

Oxford Diffraction Xcalibur, Eos, Gemini diffractometer	2644 independent reflections
Radiation source: Enhance (Cu) X-ray Source	2282 reflections with *I* > 2σ(*I*)
graphite	*R*_int_ = 0.015
Detector resolution: 16.2312 pixels mm^-1^	θ_max_ = 67.1°, θ_min_ = 4.3°
ω scans	*h* = −10→10
Absorption correction: multi-scan *CrysAlis PRO* (Oxford Diffraction, 2010)	*k* = −11→8
*T*_min_ = 0.659, *T*_max_ = 1.000	*l* = −12→11
5471 measured reflections	

### Refinement

**Table:**

Refinement on *F*^2^	Secondary atom site location: difference Fourier map
Least-squares matrix: full	Hydrogen site location: inferred from neighbouring sites
*R*[*F*^2^ > 2σ(*F*^2^)] = 0.047	H-atom parameters constrained
*wR*(*F*^2^) = 0.137	*w* = 1/[σ^2^(*F*_o_^2^) + (0.0696*P*)^2^ + 0.1724*P*] where *P* = (*F*_o_^2^ + 2*F*_c_^2^)/3
*S* = 1.05	(Δ/σ)_max_ < 0.001
2644 reflections	Δρ_max_ = 0.48 e Å^−^^3^
190 parameters	Δρ_min_ = −0.50 e Å^−^^3^
0 restraints	Extinction correction: *SHELXL*, Fc^*^=kFc[1+0.001xFc^2^λ^3^/sin(2θ)]^-1/4^
Primary atom site location: structure-invariant direct methods	Extinction coefficient: 0.0088 (14)

### Special details

**Table:**

Geometry. All e.s.d.'s (except the e.s.d. in the dihedral angle between two l.s. planes) are estimated using the full covariance matrix. The cell e.s.d.'s are taken into account individually in the estimation of e.s.d.'s in distances, angles and torsion angles; correlations between e.s.d.'s in cell parameters are only used when they are defined by crystal symmetry. An approximate (isotropic) treatment of cell e.s.d.'s is used for estimating e.s.d.'s involving l.s. planes.
Refinement. Refinement of *F*^2^ against ALL reflections. The weighted *R*-factor *wR* and goodness of fit *S* are based on *F*^2^, conventional *R*-factors *R* are based on *F*, with *F* set to zero for negative *F*^2^. The threshold expression of *F*^2^ > σ(*F*^2^) is used only for calculating *R*-factors(gt) *etc*. and is not relevant to the choice of reflections for refinement. *R*-factors based on *F*^2^ are statistically about twice as large as those based on *F*, and *R*- factors based on ALL data will be even larger.

### Fractional atomic coordinates and isotropic or equivalent isotropic displacement parameters (Å^2^)

**Table:**

	*x*	*y*	*z*	*U*_iso_*/*U*_eq_	Occ. (<1)
O1	0.42456 (15)	0.62744 (13)	0.74629 (10)	0.0489 (3)	
O2	0.70231 (17)	0.91501 (15)	0.80687 (12)	0.0603 (4)	
N1	0.16240 (18)	0.42342 (15)	0.79032 (13)	0.0462 (3)	
C1	−0.0068 (2)	0.5813 (2)	0.80579 (17)	0.0524 (4)	
H1	0.0679	0.6433	0.8878	0.063*	
C2	−0.1479 (3)	0.6089 (3)	0.7517 (2)	0.0664 (5)	
H2	−0.1677	0.6888	0.7980	0.080*	
C3	−0.2584 (3)	0.5198 (3)	0.6308 (2)	0.0721 (6)	
H3	−0.3528	0.5391	0.5950	0.087*	
C4	−0.2286 (3)	0.4014 (3)	0.5626 (2)	0.0708 (6)	
H4	−0.3028	0.3410	0.4801	0.085*	
C5	−0.0896 (2)	0.3719 (2)	0.61587 (17)	0.0579 (5)	
H5	−0.0713	0.2911	0.5693	0.070*	
C6	0.0235 (2)	0.46179 (18)	0.73834 (15)	0.0439 (4)	
C7	0.3052 (2)	0.45678 (19)	0.72467 (17)	0.0494 (4)	
H7A	0.2516	0.4097	0.6322	0.059*	
H7B	0.3766	0.4053	0.7539	0.059*	
C8	0.2421 (2)	0.4814 (2)	0.93066 (16)	0.0495 (4)	
H8A	0.2835	0.4084	0.9594	0.059*	
H8B	0.1492	0.4807	0.9743	0.059*	
C9	0.4001 (2)	0.65000 (19)	0.96899 (15)	0.0443 (4)	
C10	0.4830 (2)	0.71108 (19)	0.87442 (15)	0.0433 (4)	
C11	0.6315 (2)	0.86659 (19)	0.90761 (16)	0.0475 (4)	
C12	0.6934 (2)	0.9570 (2)	1.03462 (18)	0.0555 (4)	
H12	0.7927	1.0595	1.0567	0.067*	
C13	0.6105 (3)	0.8981 (2)	1.13087 (17)	0.0566 (4)	
C14	0.4646 (2)	0.7445 (2)	1.09664 (16)	0.0517 (4)	
H14	0.4085	0.7035	1.1599	0.062*	
C15	0.6797 (5)	1.0036 (3)	1.2695 (2)	0.0958 (6)	
H15A	0.7676	1.1113	1.2695	0.115*	0.662 (4)
H15B	0.5782	1.0117	1.2961	0.115*	0.662 (4)
H15C	0.8114	1.0521	1.2917	0.115*	0.338 (4)
H15D	0.6483	1.0908	1.2695	0.115*	0.338 (4)
C16	0.7611 (5)	0.9503 (4)	1.3628 (3)	0.0737 (8)	0.662 (4)
H16	0.8657	0.9456	1.3499	0.088*	0.662 (4)
C17	0.7103 (4)	0.9065 (3)	1.4652 (2)	0.0958 (6)	
H17A	0.6068	0.9083	1.4840	0.115*	0.662 (4)
H17B	0.7776	0.8736	1.5192	0.115*	0.662 (4)
H17C	0.8326	0.9343	1.4744	0.115*	0.338 (4)
H17D	0.6501	0.8585	1.5244	0.115*	0.338 (4)
C18	0.8505 (3)	1.0730 (2)	0.8341 (2)	0.0700 (6)	
H18A	0.8854	1.0930	0.7554	0.105*	
H18B	0.9516	1.0816	0.8959	0.105*	
H18C	0.8144	1.1509	0.8692	0.105*	
C16A	0.6213 (10)	0.9354 (8)	1.3672 (6)	0.0737 (8)	0.338 (4)
H16A	0.4995	0.9041	1.3648	0.088*	0.338 (4)

### Atomic displacement parameters (Å^2^)

**Table:**

	*U*^11^	*U*^22^	*U*^33^	*U*^12^	*U*^13^	*U*^23^
O1	0.0500 (6)	0.0476 (6)	0.0440 (6)	0.0192 (5)	0.0114 (5)	0.0092 (5)
O2	0.0589 (7)	0.0502 (7)	0.0648 (8)	0.0165 (6)	0.0224 (6)	0.0160 (6)
N1	0.0484 (7)	0.0398 (7)	0.0461 (7)	0.0182 (6)	0.0075 (6)	0.0107 (5)
C1	0.0509 (9)	0.0496 (9)	0.0533 (9)	0.0213 (7)	0.0117 (7)	0.0117 (7)
C2	0.0639 (11)	0.0742 (13)	0.0799 (14)	0.0418 (10)	0.0281 (10)	0.0305 (10)
C3	0.0525 (11)	0.1021 (16)	0.0756 (13)	0.0392 (11)	0.0188 (10)	0.0464 (12)
C4	0.0531 (10)	0.0885 (15)	0.0530 (10)	0.0197 (10)	0.0013 (8)	0.0223 (10)
C5	0.0553 (10)	0.0554 (10)	0.0493 (9)	0.0174 (8)	0.0063 (8)	0.0086 (8)
C6	0.0414 (8)	0.0380 (7)	0.0461 (8)	0.0119 (6)	0.0106 (6)	0.0142 (6)
C7	0.0531 (9)	0.0434 (8)	0.0519 (9)	0.0244 (7)	0.0114 (7)	0.0085 (7)
C8	0.0511 (9)	0.0496 (9)	0.0475 (9)	0.0219 (7)	0.0091 (7)	0.0192 (7)
C9	0.0443 (8)	0.0465 (8)	0.0451 (8)	0.0246 (7)	0.0063 (6)	0.0143 (6)
C10	0.0429 (8)	0.0445 (8)	0.0443 (8)	0.0238 (7)	0.0069 (6)	0.0102 (6)
C11	0.0465 (8)	0.0436 (8)	0.0556 (9)	0.0235 (7)	0.0114 (7)	0.0144 (7)
C12	0.0540 (10)	0.0418 (8)	0.0602 (10)	0.0184 (7)	0.0027 (8)	0.0086 (7)
C13	0.0676 (11)	0.0496 (9)	0.0498 (9)	0.0309 (8)	0.0024 (8)	0.0066 (7)
C14	0.0618 (10)	0.0556 (10)	0.0444 (9)	0.0331 (8)	0.0108 (7)	0.0159 (7)
C15	0.1375 (17)	0.0678 (10)	0.0570 (9)	0.0373 (11)	0.0030 (10)	0.0026 (8)
C16	0.0783 (19)	0.0780 (18)	0.0525 (14)	0.0396 (17)	−0.0006 (14)	−0.0091 (13)
C17	0.1375 (17)	0.0678 (10)	0.0570 (9)	0.0373 (11)	0.0030 (10)	0.0026 (8)
C18	0.0596 (11)	0.0532 (10)	0.0888 (15)	0.0155 (9)	0.0242 (10)	0.0226 (10)
C16A	0.0783 (19)	0.0780 (18)	0.0525 (14)	0.0396 (17)	−0.0006 (14)	−0.0091 (13)

### Geometric parameters (Å, °)

**Table:**

O1—C7	1.4463 (19)	C10—C11	1.404 (2)
O1—C10	1.3716 (18)	C11—C12	1.378 (2)
O2—C11	1.367 (2)	C12—H12	0.9300
O2—C18	1.423 (2)	C12—C13	1.395 (3)
N1—C6	1.427 (2)	C13—C14	1.384 (3)
N1—C7	1.432 (2)	C13—C15	1.524 (3)
N1—C8	1.463 (2)	C14—H14	0.9300
C1—H1	0.9300	C15—H15A	0.9700
C1—C2	1.384 (3)	C15—H15B	0.9700
C1—C6	1.387 (2)	C15—H15C	0.9700
C2—H2	0.9300	C15—H15D	0.9700
C2—C3	1.368 (3)	C15—C16	1.408 (4)
C3—H3	0.9300	C15—C16A	1.368 (8)
C3—C4	1.379 (3)	C16—H16	0.9300
C4—H4	0.9300	C16—C17	1.308 (4)
C4—C5	1.378 (3)	C17—H17A	0.9300
C5—H5	0.9300	C17—H17B	0.9300
C5—C6	1.388 (2)	C17—H17C	0.9300
C7—H7A	0.9700	C17—H17D	0.9300
C7—H7B	0.9700	C17—C16A	1.332 (7)
C8—H8A	0.9700	C18—H18A	0.9600
C8—H8B	0.9700	C18—H18B	0.9600
C8—C9	1.511 (2)	C18—H18C	0.9600
C9—C10	1.386 (2)	C16A—H16A	0.9300
C9—C14	1.395 (2)		
			
C10—O1—C7	113.65 (12)	C13—C14—C9	121.22 (16)
C11—O2—C18	117.67 (15)	C13—C14—H14	119.4
C6—N1—C7	115.33 (13)	C13—C15—H15A	108.3
C6—N1—C8	117.51 (13)	C13—C15—H15B	108.3
C7—N1—C8	109.17 (13)	C13—C15—H15C	107.5
C2—C1—H1	119.9	C13—C15—H15D	107.5
C2—C1—C6	120.30 (17)	H15A—C15—H15B	107.4
C6—C1—H1	119.9	H15A—C15—H15C	51.2
C1—C2—H2	119.6	H15A—C15—H15D	57.7
C3—C2—C1	120.71 (19)	H15B—C15—H15C	142.9
C3—C2—H2	119.6	H15B—C15—H15D	52.4
C2—C3—H3	120.3	H15C—C15—H15D	107.0
C2—C3—C4	119.46 (18)	C16—C15—C13	115.9 (2)
C4—C3—H3	120.3	C16—C15—H15A	108.3
C3—C4—H4	119.8	C16—C15—H15B	108.3
C5—C4—C3	120.37 (18)	C16—C15—H15C	63.0
C5—C4—H4	119.8	C16—C15—H15D	136.5
C4—C5—H5	119.7	C16A—C15—C13	119.2 (3)
C4—C5—C6	120.62 (18)	C16A—C15—H15A	132.4
C6—C5—H5	119.7	C16A—C15—H15B	61.8
C1—C6—N1	123.14 (14)	C16A—C15—H15C	107.5
C1—C6—C5	118.54 (16)	C16A—C15—H15D	107.5
C5—C6—N1	118.30 (15)	C16A—C15—C16	48.1 (3)
O1—C7—H7A	108.9	C15—C16—H16	115.5
O1—C7—H7B	108.9	C17—C16—C15	129.1 (3)
N1—C7—O1	113.26 (13)	C17—C16—H16	115.5
N1—C7—H7A	108.9	C16—C17—H17A	120.0
N1—C7—H7B	108.9	C16—C17—H17B	120.0
H7A—C7—H7B	107.7	C16—C17—H17C	70.0
N1—C8—H8A	109.1	C16—C17—H17D	167.1
N1—C8—H8B	109.1	C16—C17—C16A	50.8 (4)
N1—C8—C9	112.41 (13)	H17A—C17—H17B	120.0
H8A—C8—H8B	107.9	H17A—C17—H17C	159.0
C9—C8—H8A	109.1	H17A—C17—H17D	53.8
C9—C8—H8B	109.1	H17B—C17—H17C	53.8
C10—C9—C8	118.78 (14)	H17B—C17—H17D	67.4
C10—C9—C14	119.50 (15)	H17C—C17—H17D	120.0
C14—C9—C8	121.72 (15)	C16A—C17—H17A	69.9
O1—C10—C9	122.98 (14)	C16A—C17—H17B	167.4
O1—C10—C11	117.02 (14)	C16A—C17—H17C	120.0
C9—C10—C11	119.99 (15)	C16A—C17—H17D	120.0
O2—C11—C10	115.13 (14)	O2—C18—H18A	109.5
O2—C11—C12	125.52 (15)	O2—C18—H18B	109.5
C12—C11—C10	119.36 (16)	O2—C18—H18C	109.5
C11—C12—H12	119.3	H18A—C18—H18B	109.5
C11—C12—C13	121.45 (16)	H18A—C18—H18C	109.5
C13—C12—H12	119.3	H18B—C18—H18C	109.5
C12—C13—C15	119.89 (19)	C15—C16A—H16A	114.7
C14—C13—C12	118.48 (16)	C17—C16A—C15	130.5 (6)
C14—C13—C15	121.63 (19)	C17—C16A—H16A	114.7
C9—C14—H14	119.4		
			
O1—C10—C11—O2	−1.0 (2)	C8—C9—C14—C13	179.36 (15)
O1—C10—C11—C12	178.79 (14)	C9—C10—C11—O2	−179.74 (13)
O2—C11—C12—C13	179.05 (15)	C9—C10—C11—C12	0.1 (2)
N1—C8—C9—C10	−17.6 (2)	C10—O1—C7—N1	47.99 (18)
N1—C8—C9—C14	162.77 (14)	C10—C9—C14—C13	−0.3 (2)
C1—C2—C3—C4	0.1 (3)	C10—C11—C12—C13	−0.7 (3)
C2—C1—C6—N1	−177.76 (15)	C11—C12—C13—C14	0.9 (3)
C2—C1—C6—C5	0.4 (2)	C11—C12—C13—C15	−178.27 (19)
C2—C3—C4—C5	0.4 (3)	C12—C13—C14—C9	−0.4 (3)
C3—C4—C5—C6	−0.6 (3)	C12—C13—C15—C16	−113.3 (3)
C4—C5—C6—N1	178.38 (16)	C12—C13—C15—C16A	−167.9 (4)
C4—C5—C6—C1	0.2 (3)	C13—C15—C16—C17	−117.6 (3)
C6—N1—C7—O1	71.23 (17)	C13—C15—C16A—C17	110.4 (6)
C6—N1—C8—C9	−87.32 (17)	C14—C9—C10—O1	−178.21 (13)
C6—C1—C2—C3	−0.5 (3)	C14—C9—C10—C11	0.4 (2)
C7—O1—C10—C9	−16.4 (2)	C14—C13—C15—C16	67.6 (4)
C7—O1—C10—C11	164.92 (13)	C14—C13—C15—C16A	13.0 (5)
C7—N1—C6—C1	−114.38 (17)	C15—C13—C14—C9	178.77 (18)
C7—N1—C6—C5	67.48 (18)	C15—C16—C17—C16A	10.4 (4)
C7—N1—C8—C9	46.45 (17)	C16—C15—C16A—C17	10.5 (4)
C8—N1—C6—C1	16.6 (2)	C16—C17—C16A—C15	−10.9 (4)
C8—N1—C6—C5	−161.52 (14)	C18—O2—C11—C10	178.62 (15)
C8—N1—C7—O1	−63.64 (17)	C18—O2—C11—C12	−1.2 (2)
C8—C9—C10—O1	2.1 (2)	C16A—C15—C16—C17	−10.5 (4)
C8—C9—C10—C11	−179.22 (13)		

## References

[bb1] Chen, X.-L., Diao, X.-J. & Wu, M.-H. (2007). *Acta Cryst.* E**63**, o3580.

[bb2] Cremer, D. & Pople, J. A. (1975). *J. Am. Chem. Soc.* **97**, 1354–1358.

[bb3] Dolomanov, O. V., Bourhis, L. J., Gildea, R. J., Howard, J. A. K. & Puschmann, H. (2009). *J. Appl. Cryst.* **42**, 339–341.

[bb4] Kiskan, B., Yagci, Y., Sahmetlioglu, E. & Toppare, L. (2007). *J. Polym. Sci. Part A Polym. Chem.* **45**, 999–1006.

[bb5] Liu, Y.-L., Hsu, C.-W. & Chou, C.-I. (2007). *J. Polym. Sci. Part A Polym. Chem.* **45**, 1007–1015.

[bb6] Oxford Diffraction (2010). *CrysAlis PRO* . Oxford Diffraction Ltd, Yarnton, England.

[bb7] Ran, Q.-C. & Gu, Y. (2011). *J. Polym. Sci. Part A Polym. Chem.* **49**, 1671–1677.

[bb8] Sawaryn, C., Landfester, K. & Taden, A. (2010). *Macromolecules*, **43**, 8933–8941.

[bb9] Sheldrick, G. M. (2008). *Acta Cryst.* A**64**, 112–122. 10.1107/S0108767307043930 18156677

[bb10] Su, Y.-C., Yei, D.-R. & Chang, F.-C. (2005). *J. Appl. Polym. Sci.* **95**, 730–737.

